# Supplementary material for: Cardiometabolic Health in Relation to Lifestyle and Body Weight Changes 3–8 Years Earlier
Source: Nutrients. 2018 Dec 10;10(12):1953. doi: 10.3390/nu10121953 (PMC6315733; doi:10.3390/nu10121953)
Supplement: Supplementary file 1 [file nutrients-10-01953-s001.pdf]

Table S1. Characteristics and baseline cardiometabolic health of participants versus the non-participants.

	**Participants (N=111)**	**Non-participants (N=463)**	**P-value**
Age at baseline (years; mean; SD)	30.4 (4.1)	29.6 (4.6)	0.07
Caucasian (N; %)	105 (94.6)	397 (85.7)	0.01
Education level (N; %)			
No education or primary school (4-12 years)	1 (0.9)	26 (5.9)	0.17
Secondary education	25 (23.6)	106 (23.9)	
Intermediate Vocational Education	52 (49.1)	214 (48.3)	
Higher Vocational Education or University	28 (26.4)	97 (21.9)	
PCOS (yes; N; %)	43 (38.7)	158 (34.3)	0.38
Cardiovascular outcomes at baseline			
BMI at randomization (kg/m^2; mean; SD)	35.7 (3.0)	36.1 (3.5)	0.19
Systolic blood pressure (mmHg; mean; SD)	125.4 (12.3)	126.7 (14.3)	0.39
Diastolic blood pressure (mmHg; mean; SD)	80.4 (7.8)	79.6 (9.4)	0.41
Metabolic outcomes at baseline			
Glucose (mmol/L; mean; SD)	5.3 (0.7)	5.4 (0.7)	0.59
Insulin (pmol/L; mean; SD)	14.0 (7.2)	14.5 (8.4)	0.54
HOMA-IR (mean; SD)	0.6 (0.4)	0.6 (0.4)	0.67
Triglycerides (mmol/L; mean; SD)	1.3 (1.1)	1.3 (1.3)	0.82
Total cholesterol (mmol/L; mean; SD)	4.7 (0.9)	4.8 (0.9)	0.39
LDL-C (mmol/L; mean; SD)	3.0 (0.8)	3.1 (0.8)	0.33
HDL-C (mmol/L; mean; SD)	1.2 (0.3)	1.2 (0.3)	0.38
Metabolic syndrome at baseline (yes; N; %)	46 (51.7)	213 (55.8)	0.49
Change in dietary intake and physical activity*			
Vegetable intake (gram/day; mean; SD)	1.9 (58.7)	3.6 (66.7)	0.83
Fruit intake (gram/day; mean; SD)	25.6 (75.6)	14.4 (70.6)	0.19
Sugary drinks (glasses/day; mean; SD)	-0.4 (1.8)	-0.5 (2.5)	0.81
Savoury snacks (handful/week; mean; SD)	-1.7 (5.6)	-3.4 (8.5)	0.07
Sweet snacks (portion/week; mean; SD)	-0.1 (5.6)	-3.3 (10.2)	<0.001
Total MVPA (hour/week; mean; SD)	1.1 (12.1)	0.7 (13.6)	0.83
Change in body weight (kg; mean; SD)	-2.6 (5.1)	-2.8 (5.4)	0.78

PCOS = Polycystic Ovary Syndrome; BMI = Body Mass Index; HOMA-IR = Homeostatic Model Assessment of Insulin Resistance; LDL-C = low-density lipoproteins cholesterol; HDL-C = high-density lipoproteins cholesterol; MVPA = Moderate to Vigorous Physical Activity. * For all variables the change was calculated as preferably 6 months or otherwise 3 months minus baseline. If a higher score on the change variable is favorable or not depends on the independent variable of interest: A higher change score for vegetable intake, fruit intake, and MVPA is healthier, while a higher change score for sugary drink intake, savory and sweet snack intake, and weight change (higher means weight gain instead of weight loss) is unhealthier.
